# Clinical impact of carbon‐ion radiotherapy on hepatocellular carcinoma with Child‐Pugh B cirrhosis

**DOI:** 10.1002/cam4.6046

**Published:** 2023-05-10

**Authors:** Yuichi Hiroshima, Masaru Wakatsuki, Takashi Kaneko, Hirokazu Makishima, Naomi Nagatake Okada, Shigeo Yasuda, Hitoshi Ishikawa, Hiroshi Tsuji

**Affiliations:** ^1^ QST Hospital National Institutes for Quantum Sciences and Technology Chiba Japan; ^2^ Department of Radiation Oncology, Faculty of Medicine University of Tsukuba Tsukuba Japan; ^3^ Department of Radiation Oncology Yamagata University Faculty of Medicine Yamagata Japan; ^4^ Department of Radiology Chiba Rosai Hospital Chiba Japan

**Keywords:** carbon‐ion radiotherapy, Child‐Pugh B, hepatocellular carcinoma, radiation‐induced liver injury

## Abstract

**Background and Aims:**

Hepatocellular carcinoma (HCC) patients with Child‐Pugh (CP)‐B not eligible for surgery nor other focal therapy options due to impaired liver function, have very limited treatment options. This study aims to retrospectively investigate the toxicity and efficacy of Carbon‐ion radiotherapy (C‐ion RT) on HCC with CP‐B patients.

**Materials and Methods:**

Patients with CP‐B, no extrahepatic metastasis, and treated with C‐ion RT between May 2000 and March 2020 were retrospectively extracted and included in this study.

**Results:**

Sixty‐nine lesions of 58 patients were included. The median follow‐up duration was 20.5 (2.7–108) months. During follow‐up, recurrence was observed in 43 patients, including 2 local recurrences and 39 intrahepatic recurrences beyond the irradiation field. A grade 3 acute hepatotoxicity was observed in one patient during the observation period. No acute or late adverse event of grade ≥4 was observed. Overall survival was 80.4% and 46.0% at 1 and 2 years, respectively, and the median survival time was 22.6 months. Local control rate was 96.4% at both 1 and 2 years, and progression‐free survival was 38.6% and 6.9% at 1 and 2 years, respectively, with a median of 9.7 months.

**Conclusion:**

The C‐ion RT showed low toxicity and good local effect in patients with HCC and CP‐B. Therefore, C‐ion RT could be an appropriate treatment for patients with HCC with poor liver function.

**Lay summary**

Carbon‐ion RT may improve treatment outcome of patients with poor liver function.

## 1 INTRODUCTION

Hepatocellular carcinoma (HCC) is the most common primary cancer in the liver and in Japan, the fifth most common cause of cancer‐related deaths.^1^ HCC generally arises from chronic hepatitis/cirrhosis often caused by reasons such as hepatitis B/C, alcohol, or non‐alcoholic steatohepatitis (NASH); and in patients with impaired liver function, the progression of HCC is a major cause of death.^2^ Hepatectomy is the standard treatment for those who are fit, but many patients cannot undergo surgery for medical and anatomical reasons. In Japan, radiofrequency ablation (RFA) and transcatheter arterial chemoembolization (TACE) have been used as local therapies in addition to surgery, but the indications for RFA are limited by the size and number of lesions, while TACE is limited by liver function.^3^ There is also a risk of liver dysfunction associated with bile duct injury with RFA. There are various methods to assess liver function, but the most frequently used are Child‐Pugh (CP) score and the ALBI (Albumin‐Bilirubin) Grade. The CP score has been widely used to assess liver function.^4^, ^5^, ^6^ CP‐A, CP‐B, and CP‐C are described as having few symptoms of liver failure, intermediate liver failure, and severe liver failure, respectively. According to the Japanese guidelines for the treatment of liver cancer, local treatment, such as surgery, is indicated for CP‐A and CP‐B, while liver transplantation is recommended for CP‐C.^3^ Among other factors, liver function in CP‐B patients is known to range from tolerable to difficult to treat locally, including surgery. Other countries also have guidelines that describe similar treatment strategies.^7^, ^8^ However, to the best of our knowledge, there are no clear guidelines for treatment strategy for the liver cancer of CP‐B patient with poor liver function; thus, it is important to offer a safe and effective treatment option for each patient.

Stereotactic body radiotherapy (SBRT), a form of high‐precision photon radiotherapy, has recently become more widespread. SBRT is often used when the location of the lesion or the patient's condition makes it difficult to perform surgery or RFA. SBRT has been shown to have high local control rates and low toxicity for HCC in prospective and multicenter studies.^9^, ^10^, ^11^, ^12^ However, most of the studies have described results of treatment for patients with CP‐A and small HCC. A few studies reporting the results of SBRT for CP‐B patients have described serious adverse events and necessity of decrease in the mean liver dose according to tumor volume.^13^, ^14^ This suggests that safe treatment may be difficult for the SBRT depending on the size of the lesion and volume of the remaining normal liver.^13^, ^14^ Furthermore, local treatments, such as TACE and RFA, are highly toxic in patients with CP‐B, and thus, limiting the treatment options for patients with HCC with CP‐B.^15^

Particle radiotherapy, including carbon‐ion radiotherapy (C‐ion RT), is a radiation therapy type that uses charged particles and is clinically applied to various of cancer types.^16^, ^17^, ^18^, ^19^ It is observed that depending on the energy given by the accelerator, a maximum energy is imparted to the tissue at the end of beam range after entering the body, and the tissue at deeper areas is much less affected. Therefore C‐ion RT can offer more concentrated dose distribution than SBRT. This property could be used to safely and adequately treat patients with a poor background liver, such as those with CP‐B. This property may also be advantageous in the treatment of positions that are difficult to irradiate with SBRT, such as the caudate lobe.

The present study aimed to review C‐ion RT performed on patients with HCC with CP‐B background liver and evaluate its therapeutic efficacy and toxicity.

## 2 MATERIALS AND METHODS

### 2.1 Patients

Cases were extracted from an inhouse all‐in treatment database matching the following criteria: (1) HCC diagnosed pathologically by biopsy or imaging by computed tomography (CT)/magnetic resonance imaging (MRI); (2) presence of CP‐B liver; (3) absence of distant organ or lymph node metastasis evaluated by CT; (4) tumor not in contact with the digestive tract; (5) ≤3 irradiation fields; (6) difficulty in or refusal for receiving standard treatment; and (7) Eastern Cooperative Oncology Group performance status 0–2. The exclusion criteria were as follows: (1) previous radiation therapy for the target tumor; (2) portal hypertension with well‐developed extrahepatic portal collateral vessels; (3) difficult‐to‐treat gastric or esophageal varices, and (4) other active cancers (Figure S1). In this study, portal invasion and vessel invasion were recognized based on Japanese guideline.^20^ We considered conditions such as pre‐treatment portal hypertension to be exclusion factors in this study, as they would interfere with the post‐treatment evaluation of CP B patients.

All patients were evaluated for eligibility to receive C‐ion RT by the Ethics Committee and a Tumor Board comprising experts consisting of gastroenterologists, gastroenterological surgeons, and radiation oncologists. The participants provided informed consent for participation or had the opportunity to opt‐out of the study.

### 2.2 C‐ion RT treatment and follow‐up

Before performing CT for planning treatment, fiducial markers were inserted near the tumor in the liver under ultrasound examination. For patients with ascites in the path of the beam, diuretics would be prescribed to ensure that the ascites was gone or that the increase or decrease was stable before proceeding to treatment planning. The CT was performed at each respiratory phase as 4D‐CT. Thermoplastic sheets (Shellfitter; Kuraray), customized cradles (Moldcare; Alcare), and gating system (NIRS) were used during the treatment plan CT and treatment room to improve alignment and accuracy during treatment.^21^ Before each treatment, the bone and fiducial markers were checked against those in the treatment plan using the fluoroscope attached to the treatment device, in order to keep the sufficient positioning accuracy. If the accuracy was insufficient, CT acquisition and treatment planning were reconsidered. For sufficient and insufficient accuracy, 3 mm was used as the threshold.

The C‐ion RT method was reported previously.^22^, ^23^, ^24^ In short, the gross tumor volume (GTV) was set as the treatment plan CT that was fused with contrast‐enhanced CT/MRI, and a clinical target volume (CTV) margin of 5–10 mm was included in the GTV. If vascular invasion was suspected, a 20 mm CTV margin was set along the vascular line. An internal margin of 3–5 mm and a setup margin of 2–3 mm were included in the planning target volume (PTV).

The radiation dose of C‐ion RT was expressed as the photon equivalent dose (Gy) (i.e., relative biological effect [RBE] weighted absorbed dose) and defined as the physical dose multiplied by the C‐ion RBE.^25^ In this study, RBE‐weighted doses in C‐ion RT were expressed in Gy according to the guidelines of ICRU93. We used two or four dose fractions depending on whether an organ at risk, such as the gastrointestinal tract, is close to the lesion. The total dose and dose fraction were 45 or 48 Gy/2 fractions (fr), in other words 22.5 or 24 Gy/fr, respectively, when no organ at risk, such as the gastrointestinal (GI) tract, was in proximity, and 52.8 or 60 Gy/4 fr, in other words 13.2 or 15 Gy/fr, when it was. A fasting period of at least 3 hours before the treatment was required. If the GI tract was close to the irradiation area, a fasting period of at least 6 hours was required before the start of each treatment to improve reproducibility and reduce adverse events. A treatment plan was made to ensure that the non‐irradiated volume of the liver was at least 500 mL. The dose constraint to the GI tract was set at approximately 24 Gy of D2cc for 2 fr protocol and 30 Gy for 4 fr, to the spinal cord was set at less than 25 Gy of maximum dose, and as far as possible, the other organ at risks were not irradiated. C‐ion RT treatment planning was recently performed with the XiO‐N (ELEKTA), which uses pencil beam algorithm.^26^

After completion of C‐ion RT, patients were required to undergo follow‐up every 3 months, and CT and MRI were performed every 3–6 months to evaluate the lesions and check for recurrence. Local recurrence was defined as regrowth of the irradiated lesion after C‐ion RT by CT and MRI. Regional recurrence was defined as recurrence within the liver outside the irradiated area. Blood samples were collected during each visit. Adverse events were assessed in accordance with the Common Terminology Criteria for Adverse Events, version 4.0, and previous reports.^27^, ^28^, ^29^ Of the adverse events, the acute phase was defined as that occurring within 3 months of treatment, and late phase was defined as that occurring after 3 months of treatment.

### 2.3 Statistical analysis

The Kaplan–Meier method was used to analyze overall survival (OS), local control (LC), and progression‐free survival (PFS), and the log‐rank test was used for comparison between elements. Age, sex, and CP score were included in the analysis. The relationship between acute and late CP scores and PTV, mean irradiated liver dose (MLD), and whole liver volume was examined using t‐tests. SPSS software version 25.0 (IBM Inc.) was used for all analyses. A *p* < 0.05 was considered to be statistically significant.

### 2.4 Ethics approval

This research was conducted in accordance with the Declaration of Helsinki (1964) and the subsequent Code of Ethics, and the study design was approved by the Institutional Review Board (permit number: 20‐046).

## 3 RESULTS

### 3.1 Baseline characteristics

Between May 2000 and March 2020, 496 cases of HCC were treated with C‐ion RT, and 69 lesion of 58 patients met the criteria of this study. There were 36 men and 22 women with a median age of 71 (range, 49–84) years. Hepatitis B, hepatitis C, alcoholic hepatitis, and NASH were detected in 7, 33, 7, and 4 patients, respectively, as background liver conditions. The CP score were 7, 8, and 9 in 42 (72.4%), 13 (22.4%), and 3 (5.2%) patients, respectively. No patient received systemic therapy prior to C‐ion RT. No patient was treated at two sites at the same time, and there were 9 patients who received C‐ion RT for intrahepatic recurrence at different times. Three patients had four intrahepatic lesions, but the placement was such that treatment was possible with one irradiation field. The median PTV was 74.6 mL (range, 16.0–618.4 mL), and MLD was 8.7 Gy (range, 2.2–28.5 Gy). Other patient backgrounds are shown in Table 1.

**TABLE 1:** Patient characteristics.

		Number of patients or tumors
Age	Median 71(range 49–84)	
Sex	Male/female	36/22
Performance Status	0/1/2	43/12/3
Background Liver	B/C/ALC/NASH/others	7/33/7/4/7
Local therapy history	Yes/No	44/14
Surgical history	Yes/No	7/51
Primary or recurrence (site)	Primary/Regional rec/Local rec/Regional and Local rec	14/23/24/8
Portal vein invasion	0/1/2/3/4	61/0/4/2/2
Venous invasion	0/1/2/3/4	63/0/5/1/0
Bile duct invasion	0/1/2/3/4	65/1/1/2/0
Total number of intrahepatic tumors	1/2/3/4	52/10/3/3
Location of tumor	S 1/2/3/4/5/6/7/8	4/2/5/12/12/3/11/20
Whole liver volume (mL)	Median 1003.8 (range 567.3–2018.7)	
Size of tumor (cm)	Median 3.2 (range 0.7–13.5)	
Planning target volume (mL)	Median 74.6 (range 16.0–618.4)	
Whole liver volume—Planning target volume (mL)	Median 889.4 (range 438.9–1884.7)	
Child‐Pugh score	7/8/9	42/13/3
Ascites	None/small dose/moderate dose	41/16/1
ALBI grade	1/2a/2b/3	1/7/46/4
The reason for choosing C‐ion RT	Difficulty/refusal of other treatments	54/4
Total dose (Gy)	45/48/52.8/60	9/24/27/9
Mean Liver dose (Gy)	Median 8.7 (range 2.2–28.5)	
AFP (ng/mL)	Median 49.6 (2.7–100,270)	
DCP (mAU/mL)	Median 66 (11–207,600)	

Abbreviations: AFP, alpha fetoprotein; ALC, alcoholic hepatitis; Background Liver B, hepatitis B; C, hepatitis C; DCP, Des‐gamma‐carboxy prothrombin; Gy, Gray; NASH, non‐alcoholic steatohepatitis, RBE, relative biological effectiveness; rec, recurrence.

### 3.2 Treatment outcome, toxicity, and prognostic factor

The median follow‐up period was 20.5 (range, 2.7–108) months. There were 45 deaths during the study period. Twenty‐eight, 10, 7, and 13 deaths were due to cancer, liver failure, other causes, and unknown causes, respectively. Patients who died from the progression of their current cirrhosis without developing radiation‐induced liver disease (RILD) and unrelated to direct liver damage from C‐ion RT are defined here as death from liver failure. The median follow‐up period focused only on survivors, which was 22.4 (range, 4.0–74.1) months.

During follow‐up, recurrence was observed in 43 patients, including 2 local recurrences and 39 intrahepatic recurrences out of the irradiation field. Among the recurrent cases, 2 patients were treated by RFA, 9 patients were treated by C‐ion RT, 2 patients were treated by palliative photon RT, and the rest were in best supportive care. Reasons for not performing C‐ion RT again for recurrent lesions included ADL, financial issues, and distance and time constraints for hospital visits.

**FIGURE 1:**
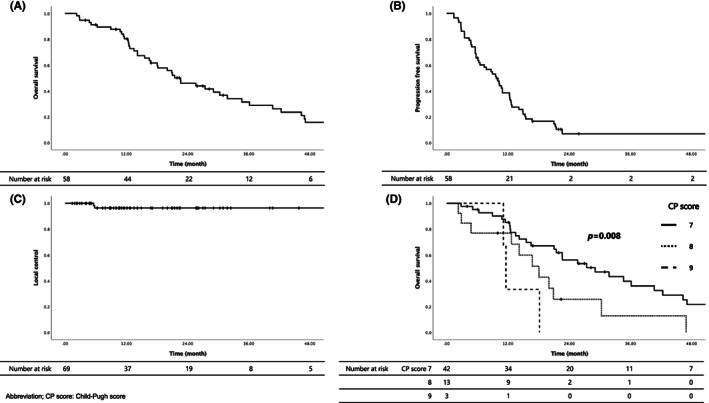
Outcomes of hepatocellular carcinoma treated with carbon‐ion radiotherapy for (A) overall survival, (B) progression‐free survival, (C) local control rate, and (D) overall survival examined by pre‐treatment Child‐Pugh score.

OS was 80.4% (95% confidence interval [CI]: 70.0%–90.8%) and 46.0% (95% CI: 32.5%–59.5%) at 1 and 2 years, respectively, and the median survival time (MST) was 22.6 months. LC was 96.4% (95% CI: 91.5%–100%) at both 1 and 2 years, and PFS was 38.6% (95% CI: 25.9%–51.3%) and 6.9% (95% CI: 0%–14.7%) at 1 and 2 years, respectively, with a median 9.7 months. Kaplan–Meier survival curves for each are shown in Figure 1.

Influences of prognostic factors of patient backgrounds, such as age, sex, and CP score on OS and PFS were analyzed; significant differences in OS were observed in ascites, CP score and alpha fetoprotein (AFP) (*p* < 0.001, *p* = 0.008, and *p* = 0.028, respectively). In PFS, there were significant differences in ascites, the liver background and total dose (*p* < 0.001, *p* = 0.014, and *p* = 0.037, respectively). The results are presented in Table 2. The Kaplan–Meier OS for the CP score is shown in Figure 1d.

**TABLE 2:** Results of univariate analysis.

	*n*	OS				PFS				LC			
		1 year (%)	2 years (%)	Median (months)	*p* value	1 year (%)	2 years (%)	Median (months)	*p* value	1 year (%)	2 years (%)	Median (months)	*p* value
Sex													
Male	36	80.0	45.7	21.5	0.866	38.4	8.9	9.6	0.658	100	100	NR	0.048
Female	22	81.1	46.5	22.7		39.0	6.5	10.3		89.5	89.5	NR	
Performance Status													
0	43	80.6	42.8	21.5	0.74	38.1	6.1	9.7	0.878	94.7	94.7	NR	0.341
1–2	15	80.0	53.3	25.6		40.0	6.7	10.3		100	100	NR	
Age													
<70	30	86.2	56.2	27.4	0.919	32.3	3.6	7.8	0.026	96.3	96.3	NR	0.990
70≤	28	74.4	35.3	18.1		45.0	11.4	10.3		96.4	96.4	NR	
Background liver													
B,C	40	79.5	39.7	20.9	0.128	32.9	0	9.6	0.014	97.5	97.5	NR	0.454
Others	18	82.4	61.0	29.1		50.0	21.6	9.7		93.3	93.3	NR	
Primary or recurrence													
Primary	14	92.3	47.5	21.5	0.915	28.6	10.7	7.4	0.873	83.3	83.3	NR	0.006
Recurrence	44	76.7	45.3	22.6		41.9	6.6	10.0		100	100	NR	
MVI													
With	13	75.5	39.2	20.0	0.479	30.8	0	5.6	0.217	88.9	88.9	NR	0.180
Without	45	81.8	47.6	22.7		40.9	8.0	10.3		97.8	97.8	NR	
Size of tumor													
<3 cm	20	85.0	54.5	25.6	0.642	35.0	5.0	7.8	0.524	100	100	NR	0.245
3 cm≤	38	78.0	40.9	21.0		40.6	9.0	9.7		93.9	93.9	NR	
Albumin (g/dL)													
<3.5	41	75.0	41.8	20.0	0.534	37.5	12.9	10.7	0.884	96.9	96.9	NR	0.600
3.5≤	17	93.8	56.3	27.4		41.2	0	9.7		93.8	93.8	NR	
Ascites													
With	17	68.3	6.8	13.5	<0.001	14.7	0	4.8	<0.001	90.9	90.9	NR	0.341
Without	41	85.2	61.0	30.3		48.2	9.5	10.9		97.3	97.3	NR	
Total dose (Gy)													
45	8	87.5	50	22.6	0.602	12.5	0	4.9	0.037	100	100	NR	0.912
48	20	79.4	47.7	22.7		44.0	13.2	10.0		95.0	95.0	NR	
52.8	25	80.0	44.0	21.0		44.0	4.0	10.7		95.8	95.8	NR	
60	5	75.0	37.5	12.3		30.0	0	6.5		100	100	NR	
Mean Liver Dose (cGy)													
<850	30	72.0	38.6	20.9	0.257	35.1	4.7	9.7	0.543	91.3	91.3	NR	0.136
850≤	28	89.0	53.1	25.6		42.3	7.7	9.6		100	100	NR	
AFP (ng/mL)													
<50	30	79.5	56.0	36.1	0.028	49.0	8.2	10.9	0.081	100	100	NR	0.146
50≤	28	81.5	34.9	18.1		27.5	3.9	7.8		92.6	92.6	NR	
DCP (mAU/mL)													
<50	27	74.1	50.7	25.6	0.755	40.7	0	10.0	0.844	100	100	NR	0.146
50≤	31	86.6	41.1	21.0		36.7	11.0	9.7		92.6	92.6	NR	
CP score													
7	42	85.2	56.1	29.1	0.008	37.0	7.5	9.6	0.255	94.6	94.6	NR	0.611
8	13	76.9	25.6	18.1		52.7	8.8	12.6		100	100	NR	
9	3	33.3	0	11.6		0	0	4.7		100	100	NR	
ALBI grade													
1	1	100	NA	16.5	0.1	0	0	5.6	0.701	0	0	NA	<0.001
2a	7	100	66.7	47.0		28.6	0	10.7		100	100	NR	
2b	46	82.3	46.1	22.6		42.4	7.3	9.7		97.7	97.7	NR	
3	4	25.0	0	11.1		25.0	0	4.7		100	100	NR	

Abbreviations: AFP, alpha fetoprotein; Background Liver B, hepatitis B; C, hepatitis C; CP score, Child‐Pugh score; DCP, Des‐gamma‐carboxy prothrombin; Gy, Gray; LC, local control rate; MVI, macrovascular invasion; *n*, number of patients; NA, not applicable; NR, not reached; OS, overall survival; PFS, progression‐free survival; RBE, relative biological effectiveness.

Regarding hepatotoxicity, a grade 3 acute adverse event was observed in one patient during the observation period but did not meet RILD criteria.^30^, ^31^ No acute or late adverse events of grade ≥4 was observed. Of the patients who completed the examination, CP score increased in the acute phase by 1 and 2 in 11 and 3 patients, respectively. In the late phase, the CP score increased by 1, 2, and 3 in 21, 1, and 1 patient, respectively (Table 3). No RILD was observed in either the acute or late phase. The relationship between MLD and exacerbation of CP score was examined, and a significant correlation was found for the acute phase, but not for the late phase (*p* = 0.031 and 0.206, respectively). On the other hand, there was no significant correlation between whole liver volume and adverse events.

**TABLE 3:** Change in Child‐Pugh score.

Pre‐treatment CP score	Acute phase						Late phase							Sum
	−2	−1	0	+1	+2	+3	−2	−1	0	+1	+2	+3	Lost	
7	2	5	22	11	2	0	3	6	13	14	1	1	4	42
8	2	4	6	0	1	0	1	1	3	5	0	0	3	13
9	0	0	3	0	0	0	0	0	1	2	0	0	0	3

Abbreviation: CP score, Child‐Pugh score.

**FIGURE 2:**
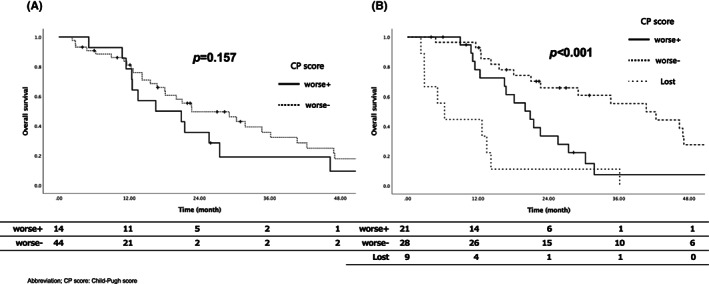
Overall survival rate compared with the presence or absence of worsening of Child‐Pugh score after treatment for (A) acute phase and (B) late phase.

We also examined the relationship between post‐treatment progression of CP score and OS. Worsening of the CP score in the acute phase was not significantly associated with shorter OS (*p* = 0.157). However, a worsening of the CP score in the late phase was significantly associated with OS (*p* < 0.001) (shown in Figure 2).

### 3.3 Examples of actual treatment

**FIGURE 3:**
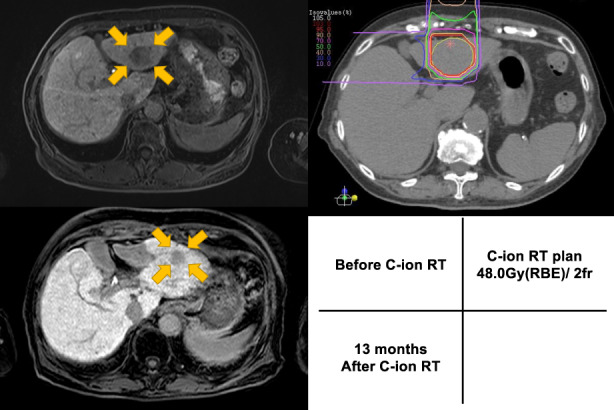
Case presentation: hepatocellular carcinoma of EOB‐MRI image before carbon‐ion radiotherapy, dose distribution of carbon‐ion radiotherapy, and EOB‐MRI image 13 months after treatment are shown.

Figure 3 shows an actual case of C‐ion RT. The patient was an 83‐year‐old woman with a CP score of 7 and B at the beginning of the treatment. A lesion was found in the S3 and was irradiated with 48 Gy (RBE)/2 fr of the C‐ion RT. Thirteen months after treatment, MRI showed obvious shrinkage of the tumor without sign of recurrence. Subsequent follow‐up revealed no change in the CP score.

## 4 DISCUSSION/CONCLUSION

The present study describes the results of C‐ion RT for HCC with CP‐B impaired liver function. To the best of our knowledge, this study has reported the largest number of cases under similar conditions.

To date, several therapeutic outcomes of CP‐B for HCC have been reported. One of the largest of these has been reported by the National Database (NDB) in Japan for liver cancer.^32^ In this study, the 2‐year OS of HCC patients with CP‐B after surgery, RFA, and TACE were reported to be 72.7%, 77.6%, and 55.8%, respectively. However, this was the outcome of patients with HCC who could safely undergo the respective local treatments; and as many of the cases in our study were relapses after receiving other treatments, it is difficult to compare correctly because outcomes of NDB were evaluates the time from initial treatment to event occurrence. In Japan, surgery, RFA, and TACE are frequently selected as the first standard treatment, and when these are difficult to be administered, radiotherapy including SBRT is selected.^33^ In addition, the Japanese guidelines for the treatment of HCC includes postoperative liver function as a factor involved in improving postoperative outcomes; this suggests that it is very important to preserve liver function after local treatment.^33^ Thus, many of cases treated with C‐ion RT were difficult to undergo other local therapies due to tumor location or liver function. Therefore, patients undergoing C‐ion RT tend to have a worse liver background and condition, leading to poor prognoses than patients receiving standard treatment.

A review of C‐ion RT outcomes for HCC shows that CP‐B has a significantly poorer prognosis than CP‐A.^22^ As for the comparison between C‐ion RT and other TACE or RFA for CP‐B patients, we cannot fully compare the results because the patients who underwent C‐ion RT are basically those who are difficult to treat with other therapies. However, since C‐ion RT has shown better results in the comparison of C‐ion RT and TACE in propensity score matching in a form not limited to CP‐B, it is expected for CP‐B as well.^34^ The comparison of propensity score matching between C‐ion RT and RFA showed similar results, which is also expected to be effective for CP‐B.^35^ Based on these points, C‐ion RT has the potential to produce therapeutic outcomes comparable to those of existing local therapies.

The results of SBRT for HCC with CP‐B have been reported by several groups. Culleton et al. performed SBRT on 29 patients with CP scores of 7–10, including 20 patients with a CP score of 7.^14^ Approximately 30 Gy was irradiated in 6 fr, and the MST was 7.9 months. This study indicated that the MST with CP score 7 was 9.9 months, while that of CP score ≥8 was significantly worse at 2.8 months. Jackson et al. reported outcomes in 80 patients with CP‐B treated with SBRT.^13^ The overall MST was 17.1 months, and the LC was 92% and 81% at 1 and 2 years, respectively. Moreover, the study found a correlation between the worsening of CP score and MLD and estimated that the CP score worsened by 1 when the MLD exceeded 11 Gy.^13^ In general, the larger PTV, the higher MLD, and the larger the tumor, the more likely it is that the CP score will worsen. However, a dosimetric study reported that the dose distribution was improved by using C‐ion RT.^36^ In this study, MLD remained low, and the frequency of adverse events was also low, despite the inclusion of relatively large HCC. This study showed that CP decline is not related to OS in the acute phase, but in the late phase is related. Since the liver has great resilience in organ function, it is thought that even if CP declines in the acute phase, the liver outside the irradiated field will provide compensatory power and CP will improve to some extent. On the other hand, if the CP is further impaired, compensation will not be effective and will lead to permanent CP deterioration. We believe that this may result in a decline in OS. These suggest that if HCC is treated with C‐ion RT, which has better dose distribution than SBRT and can be administered at sufficient doses to meet HCC radiosensitivity, without increasing MLD, a good LC rate can be expected while preventing deterioration of liver function.

Additionally, the results of the present study suggest that worsening of the CP score leads to worsening of survival rate, and thus, reducing the volume of the liver irradiated outside the target region is also important in terms of improving survival after C‐ion RT. Because of its pathogenesis, HCC is a disease with a high potential for repeated intrahepatic recurrence, and multiple treatments are often required. Although several treatments have been tried in this study for recurrence after C‐ion RT, few treatment options were considered safe due to low background liver function, and few cases were treated aggressively and radically. A threshold for confidently and safely performing C‐ion RT on such cases is a very important topic. Therefore, a future task is to predict outcomes based on the volume of the unirradiated liver and pre‐treatment liver function.

As for systemic therapy, several studies have evaluated the use of sorafenib; of which, some have reported CP‐B outcomes and toxicity. The GIDEON study, which included 368 patients with CP‐B, reported an MST of 5 months.^37^ Additionally, serious adverse events of grade ≥3 were more frequently reported in patients with CP‐B than in those with CP‐A; and in patients with CP‐B, 8 and 9 were more frequent than 7, suggesting that systemic therapy has a high risk of developing serious adverse events for patients with poor liver function. The results of this study suggest that C‐ion RT can be safely performed and may be preferred even in CP‐B patients, depending on the remaining liver function.

The survival rate in patients with liver failure has been improved due to several factors, including improvements in the treatment of hepatitis virus infections and the emergence of new hepatoprotective drugs. The Japanese NDB data suggest that survival rates have improved, possibly due to supportive care and improved treatments.^32^ Due to a long inclusion period and small number of patients in the present study, we were unable to examine the impact of pharmaceutical treatment advances. However, improvement in the prognosis of patients with CP‐B cirrhosis than that in previous years suggests a significant preference to local treatment for liver conservation. Therefore, C‐ion RT is considered to be an effective treatment method that can provide a good LC rate and minimize the effects of radiations on the liver, particularly in case the liver function can be improved by recent pharmaceutical therapy.

There are limitations to the present study: retrospective, long inclusion period, and various background liver states. However, to the best of our knowledge, there are no reports on treating a large number of patients with HCC with CP‐B using C‐ion RT; and as we report that the treatment appears to be safe, C‐ion RT could be an option for patients with impaired liver function. However, the results for CP 9 cases are not satisfactory, and due to the high risk of liver failure, we believe that the indication should be cautious and only those cases in which the dose to the liver is minimal should be applied. In addition, it is currently difficult to directly compare the outcomes of C‐ion RT with other local treatments for HCC patients with CP‐B. In the future, prospective studies focusing on CP‐B with a large number of patients collected in collaboration with multiple centers and comparisons using propensity score matching are needed. Besides, for dose–volume–histogram analysis, it is necessary to define parameters for C‐ion RT, because unlike conventional radiotherapy, C‐ion RT can be performed with fewer gates and with a little affecting the surrounding liver. This point will need to be studied in detail in the future with the addition of the CP‐A patient group.

In conclusion, this study describes the treatment outcome and toxicity of C‐ion RT in patients with HCC with CP‐B. C‐ion RT appeared to be minimally toxic and demonstrated favorable efficacy, providing a potential treatment option for those with impaired liver function suffering HCC.

## AUTHOR CONTRIBUTIONS

**Yuichi Hiroshima:** Conceptualization (lead); data curation (lead); formal analysis (lead); investigation (lead); methodology (lead); visualization (lead); writing – original draft (lead). **Masaru Wakatsuki:** Conceptualization (supporting); supervision (equal); writing – review and editing (equal). **Takashi Kaneko:** Formal analysis (supporting); resources (equal). **Hirokazu Makishima:** Investigation (supporting); resources (supporting); writing – review and editing (supporting). **Naomi Nagatake Okada:** Resources (supporting). **Shigeo Yasuda:** Resources (supporting). **Hitoshi Ishikawa:** Supervision (equal). **Hiroshi Tsuji:** Project administration (lead).

## FUNDING INFORMATION

None.

## CONFLICT OF INTEREST STATEMENT

The authors have no conflicts of interest to declare.

## Supporting information


Figure S1

## Data Availability

The data that support the findings of this study are available from the corresponding author upon reasonable request.

## References

[1] Cancer Information Service NCC, Japan (vital statistics of Japan) . Cancer Registry and Statistics. Accessed December 5, 2021. https://ganjoho.jp/reg_stat/statistics/dl/index.html

[2] Fattovich G , Stroffolini T , Zagni I , Donato F . Hepatocellular carcinoma in cirrhosis: incidence and risk factors. Gastroenterology. 2004;127(5 Suppl 1):S35‐S50. 15508101 10.1053/j.gastro.2004.09.014

[3] Kokudo N , Takemura N , Hasegawa K , et al. Clinical practice guidelines for hepatocellular carcinoma: the Japan Society of Hepatology 2017 (4th JSH‐HCC guidelines) 2019 update. Hepatol Res. 2019;49(10):1109‐1113. 31336394 10.1111/hepr.13411

[4] D'Amico G , Garcia‐Tsao G , Pagliaro L . Natural history and prognostic indicators of survival in cirrhosis: a systematic review of 118 studies. J Hepatol. 2006;44(1):217‐231. 16298014 10.1016/j.jhep.2005.10.013

[5] Pugh RN , Murray‐Lyon IM , Dawson JL , Pietroni MC , Williams R . Transection of the oesophagus for bleeding oesophageal varices. Br J Surg. 1973;60(8):646‐649. 4541913 10.1002/bjs.1800600817

[6] Child CG , Turcotte JG . Surgery and portal hypertension. Major Probl Clin Surg. 1964;1:1‐85. 4950264

[7] Network NCC . NCCN Clinical Practice Guidelines in Oncology Hepatocellular Carcinoma Version 1. 2023 Accessed April 11, 2023. https://www.nccn.org/professionals/physician_gls/pdf/hcc.pdf

[8] Vogel A , Cervantes A , Chau I , et al. Hepatocellular carcinoma: ESMO clinical practice guidelines for diagnosis, treatment and follow‐up. Ann Oncol. 2018;29(Suppl 4):iv238‐iv255. 30285213 10.1093/annonc/mdy308

[9] Kimura T , Takeda A , Sanuki N , et al. Multicenter prospective study of stereotactic body radiotherapy for previously untreated solitary primary hepatocellular carcinoma: the STRSPH study. Hepatol Res. 2021;51(4):461‐471. 33217113 10.1111/hepr.13595

[10] Yoon SM , Kim SY , Lim YS , et al. Stereotactic body radiation therapy for small (≤5 cm) hepatocellular carcinoma not amenable to curative treatment: results of a single‐arm, phase II clinical trial. Clin Mol Hepatol. 2020;26(4):506‐515. 32646200 10.3350/cmh.2020.0038 PMC7641557

[11] Jang WI , Bae SH , Kim MS , et al. A phase 2 multicenter study of stereotactic body radiotherapy for hepatocellular carcinoma: safety and efficacy. Cancer. 2020;126(2):363‐372. 31747476 10.1002/cncr.32502

[12] Durand‐Labrunie J , Baumann AS , Ayav A , et al. Curative irradiation treatment of hepatocellular carcinoma: a multicenter phase 2 trial. Int J Radiat Oncol Biol Phys. 2020;107(1):116‐125. 32001057 10.1016/j.ijrobp.2019.12.004

[13] Jackson WC , Tang M , Maurino C , et al. Individualized adaptive radiation therapy allows for safe treatment of hepatocellular carcinoma in patients with Child‐Turcotte‐Pugh B liver disease. Int J Radiat Oncol Biol Phys. 2021;109(1):212‐219. 32853708 10.1016/j.ijrobp.2020.08.046 PMC7736252

[14] Culleton S , Jiang H , Haddad CR , et al. Outcomes following definitive stereotactic body radiotherapy for patients with Child‐Pugh B or C hepatocellular carcinoma. Radiother Oncol. 2014;111(3):412‐417. 24906626 10.1016/j.radonc.2014.05.002

[15] Granito A , Bolondi L . Non‐transplant therapies for patients with hepatocellular carcinoma and Child‐Pugh‐Turcotte class B cirrhosis. Lancet Oncol. 2017;18(2):e101‐e112. 28214411 10.1016/S1470-2045(16)30569-1

[16] Yasuda S , Kato H , Imada H , et al. Long‐term results of high‐dose 2‐fraction carbon ion radiation therapy for hepatocellular carcinoma. Adv Radiat Oncol. 2020;5(2):196‐203. 32280819 10.1016/j.adro.2019.09.007 PMC7136623

[17] Shinoto M , Yamada S , Okamoto M , et al. Carbon‐ion radiotherapy for locally recurrent rectal cancer: Japan carbon‐ion radiation oncology study group (J‐CROS) study 1404 rectum. Radiother Oncol. 2019;132:236‐240. 30360998 10.1016/j.radonc.2018.10.007

[18] Kawashiro S , Yamada S , Okamoto M , et al. Multi‐institutional study of carbon‐ion radiotherapy for locally advanced pancreatic cancer: Japan carbon‐ion radiation oncology study group (J‐CROS) study 1403 pancreas. Int J Radiat Oncol Biol Phys. 2018;101(5):1212‐1221. 29907490 10.1016/j.ijrobp.2018.04.057

[19] Shinoto M , Yamada S , Terashima K , et al. Carbon ion radiation therapy with concurrent gemcitabine for patients with locally advanced pancreatic cancer. Int J Radiat Oncol Biol Phys. 2016;95(1):498‐504. 26883565 10.1016/j.ijrobp.2015.12.362

[20] Japan TLCSGo . The General Rules for the Clinical and Pathological Study of Primary Liver Cancer. Kanehara; 2019.

[21] Minohara S , Kanai T , Endo M , Noda K , Kanazawa M . Respiratory gated irradiation system for heavy‐ion radiotherapy. Int J Radiat Oncol Biol Phys. 2000;47(4):1097‐1103. 10863083 10.1016/s0360-3016(00)00524-1

[22] Kasuya G , Kato H , Yasuda S , et al. Progressive hypofractionated carbon‐ion radiotherapy for hepatocellular carcinoma: combined analyses of 2 prospective trials. Cancer. 2017;123(20):3955‐3965. 28662297 10.1002/cncr.30816 PMC5655922

[23] Imada H , Kato H , Yasuda S , et al. Comparison of efficacy and toxicity of short‐course carbon ion radiotherapy for hepatocellular carcinoma depending on their proximity to the porta hepatis. Radiother Oncol. 2010;96(2):231‐235. 20579756 10.1016/j.radonc.2010.05.019

[24] Kato H , Tsujii H , Miyamoto T , et al. Results of the first prospective study of carbon ion radiotherapy for hepatocellular carcinoma with liver cirrhosis. Int J Radiat Oncol Biol Phys. 2004;59(5):1468‐1476. 15275734 10.1016/j.ijrobp.2004.01.032

[25] Inaniwa T , Kanematsu N , Matsufuji N , et al. Reformulation of a clinical‐dose system for carbon‐ion radiotherapy treatment planning at the National Institute of Radiological Sciences, Japan. Phys Med Biol. 2015;60(8):3271‐3286. 25826534 10.1088/0031-9155/60/8/3271

[26] Kanematsu N . Dose calculation algorithm of fast fine‐heterogeneity correction for heavy charged particle radiotherapy. Phys Med. 2011;27(2):97‐102. 20579913 10.1016/j.ejmp.2010.05.001

[27] Health UDo, Services H . Common Terminology Criteria for Adverse Events (CTCAE) Version 4.0. National Institutes of Health, National Cancer Institute. 2009;4(3).

[28] Trotti A , Colevas AD , Setser A , et al. CTCAE v3. 0: development of a comprehensive grading system for the adverse effects of cancer treatment. Seminars in Radiation Oncology. Vol 13. Elsevier; 2003:176‐181. 12903007 10.1016/S1053-4296(03)00031-6

[29] Trotti A , Byhardt R , Stetz J , et al. Common toxicity criteria: version 2.0. An improved reference for grading the acute effects of cancer treatment: impact on radiotherapy. Int J Radiat Oncol Biol Phys. 2000;47(1):13‐47. 10758303 10.1016/s0360-3016(99)00559-3

[30] Guha C , Kavanagh BD . Hepatic radiation toxicity: avoidance and amelioration. Semin Radiat Oncol. 2011;21(4):256‐263. 21939854 10.1016/j.semradonc.2011.05.003 PMC3434677

[31] Cheng JC , Wu JK , Huang CM , et al. Radiation‐induced liver disease after three‐dimensional conformal radiotherapy for patients with hepatocellular carcinoma: dosimetric analysis and implication. Int J Radiat Oncol Biol Phys. 2002;54(1):156‐162. 12182986 10.1016/s0360-3016(02)02915-2

[32] Kudo M , Izumi N , Kubo S , et al. Report of the 20th nationwide follow‐up survey of primary liver cancer in Japan. Hepatol Res. 2020;50(1):15‐46. 31655492 10.1111/hepr.13438 PMC7003938

[33] Kudo M , Kawamura Y , Hasegawa K , et al. Management of hepatocellular carcinoma in Japan: JSH consensus statements and recommendations 2021 update. Liver Cancer. 2021;10(3):181‐223. 34239808 10.1159/000514174 PMC8237791

[34] Shiba S , Shibuya K , Katoh H , et al. A comparison of carbon ion radiotherapy and transarterial chemoembolization treatment outcomes for single hepatocellular carcinoma: a propensity score matching study. Radiat Oncol. 2019;14(1):137. 31375120 10.1186/s13014-019-1347-4 PMC6679447

[35] Fujita N , Kanogawa N , Makishima H , et al. Carbon‐ion radiotherapy versus radiofrequency ablation as initial treatment for early‐stage hepatocellular carcinoma. Hepatol Res. 2022;52(12):1060‐1071. 35951438 10.1111/hepr.13827

[36] Shiba S , Shibuya K , Kawashima M , et al. Comparison of dose distributions when using carbon ion radiotherapy versus intensity‐modulated radiotherapy for hepatocellular carcinoma with macroscopic vascular invasion: a retrospective analysis. Anticancer Res. 2020;40(1):459‐464. 31892601 10.21873/anticanres.13974

[37] Marrero JA , Kudo M , Venook AP , et al. Observational registry of sorafenib use in clinical practice across Child‐Pugh subgroups: the GIDEON study. J Hepatol. 2016;65(6):1140‐1147. 27469901 10.1016/j.jhep.2016.07.020

