# Supplementary figures and images for: Clinical impact of carbon‐ion radiotherapy on hepatocellular carcinoma with Child‐Pugh B cirrhosis
Source: Cancer Med. 2023 May 10;12(13):14004–14. doi: 10.1002/cam4.6046 (PMC10358263; doi:10.1002/cam4.6046)

Supplementally Figure 1

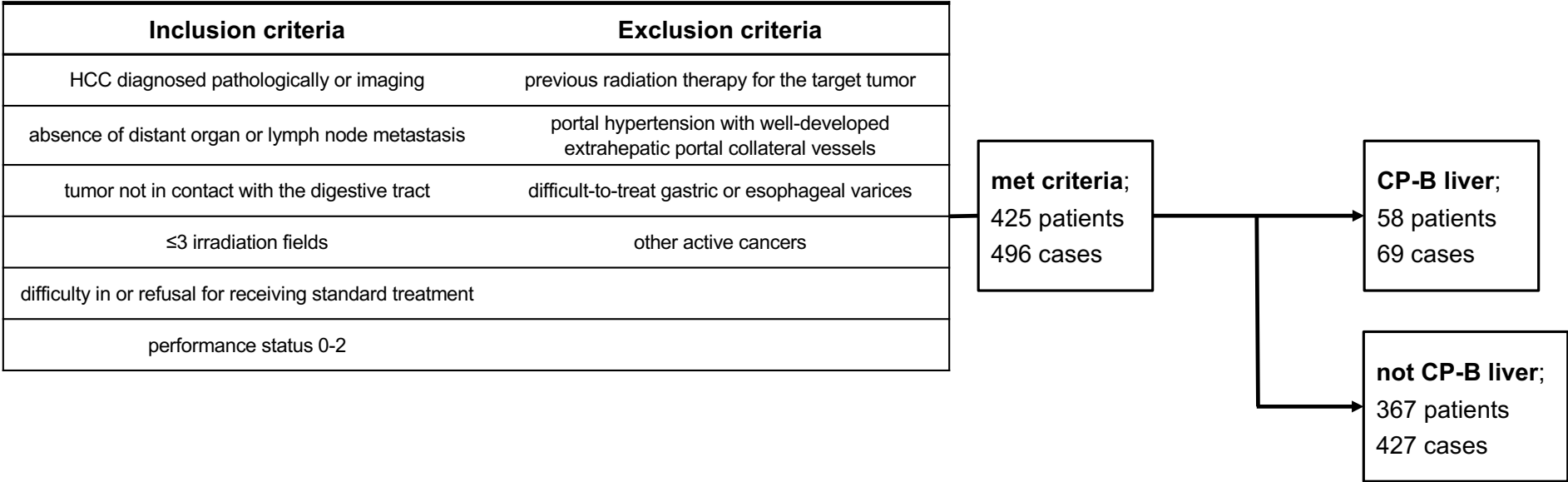

Supplement: Supplementary file 1 — Figure S1 [file CAM4-12-14004-s001.pdf]
